# Investigation of Ionic Polymers’ Stabilizing and Flocculating Properties in Dispersed Activated Carbons Systems

**DOI:** 10.3390/ma17030693

**Published:** 2024-02-01

**Authors:** Marlena Gęca, Małgorzata Wiśniewska, Piotr Nowicki

**Affiliations:** 1 Department of Radiochemistry and Environmental Chemistry, Institute of Chemical Sciences, Faculty of Chemistry, Maria Curie-Sklodowska University in Lublin, M. Curie-Sklodowska Sq. 3, 20-031 Lublin, Poland; 2 Department of Applied Chemistry, Faculty of Chemistry, Adam Mickiewicz University in Poznań, Uniwersytetu Poznańskiego 8, 61-614 Poznań, Poland; piotrnow@amu.edu.pl

**Keywords:** activated carbons, stabilization–flocculation properties, poly(acrylic acid), polyethyleneimine, binary polymer solution

## Abstract

Activated carbons obtained via the thermochemical treatment of lemon balm and mint herbs were applied for ionic polymers adsorption, which directly affects the stability of these types of aqueous suspensions. The examined carbonaceous materials were characterized by well-developed specific surface area (approximately 1000 m^2^/g) and mesoporous structure. The adsorbed amounts of anionic poly(acrylic acid) and cationic polyethyleneimine from one-component solutions reached significant levels, but the efficiency of adsorption of these compounds from binary solutions slightly decreased. Moreover, the ionic polymers showed stabilizing properties towards the activated carbons suspensions. For both adsorbents, the most stable suspensions were systems containing both types of polymeric macromolecules with different ionic characters. This was due to the occurrence of electrosteric and depletion stabilization mechanisms. Furthermore, the zeta potential and size of particle aggregates were also influenced by the presence of polymers in the aqueous suspensions of activated carbons.

## 1. Introduction

In the field of environmental remediation and water treatment, the effective removal of pollutants from aqueous media remains a critical challenge. Activated carbons, thanks to their large surface area and polydisperse porous structure, have emerged as versatile adsorbents for a diverse range of contaminants [1,2]. The flocculation process involving polymeric bridges formation between dispersed particles is important in water treatment technologies. Understanding the various mechanisms of highly-dispersed suspensions destabilization is essential for the efficient purification of aqueous solutions [3,4,5].

Ionic polymers, with their unique physicochemical properties, can affect the stability of activated carbons suspensions. These polymers, characterized by the presence of charged functional groups along their chains, have demonstrated the ability to interact with dispersed particles and influence their stability and aggregation [6]. These macromolecules’ influence on suspension stability is based on a few mechanisms: steric, electrosteric and depletion stabilization [7,8]. Steric stabilization results from polymer adsorption on the solid surface, which reduces the Van der Waals attraction between particles [9]. The electrosteric mechanism is a combination of electrostatic and steric stabilization. Polymeric adsorption layers lead to a decrease in the electrostatic and Van der Waals flocculation forces [10]. On the other hand, unadsorbed polymers chains remaining in the solution cause suppression of the depletion attraction, or depletion stabilization [11].

Poly(acrylic acid) (PAA) finds applications in many sectors, including, but not limited to, drug delivery, adhesives, textiles and biomedical engineering. It is commonly used to produce hydrogel drug-releasing systems [12,13]. The presence of carboxyl groups in PAA macromolecules enables the direct functionalisation of hydrogels via chemical and physical cross-linking [14]. In turn, polyethyleneimine (PEI) is a cationic polymer characterized by a high density of amine functional groups. This imparts PEI molecules’ unique properties, which have attracted significant attention in the fields of materials science, biotechnology and beyond. Additionally, this structure of PEI chains enables the formation of cross-links [15,16]. The possibility of adopting many different conformations on the surface of solid particles by adsorbed chains of ionic polymers has a strong impact on the stability of dispersed systems in which they occur.

In the present study, the poly(acrylic acid) and polyethyleneimine solutions, as well as activated carbons obtained from lemon balm and mint herbs, were used as components of aqueous suspensions. The zeta potential and size of aggregates of carbonaceous materials in solutions without and in the presence of single and binary polymer adsorbates were determined. The influence of polymers in one- and two-component systems on the stability of activated carbon suspensions was checked. Moreover, the impact of PAA and PEI on their mutual adsorption and the structure of mixed adsorption layers were also studied. The adsorption of ionic polymers on an activated carbon surface is rarely studied, while the mechanisms of processes that occur at the solid–solution interface are almost never investigated. However, with industrial development, the amounts of various types of macromolecular compounds in the natural environment are constantly increasing. They can disturb the natural balance and interfere with the wastewater treatment process. For this reason, there is a constant need to investigate the behaviour of such colloidal systems in order to develop more efficient techniques to separate these substances from aqueous solutions.

## 2. Materials and Methods

### 2.1. Materials

Activated carbons (AC) obtained from lemon balm (LB) and mint (MT) herbs were used in this study. The plant material was impregnated in a H_3_PO_4_ solution for 24 h and then subjected to a two-stage heating program (200 °C and 500 °C) for adsorbent preparation. The detailed procedure for adsorbents preparation was described in our previous paper [17]. The textural characterization of the activated carbons was based on nitrogen adsorption–desorption isotherms using the Brunauer–Emmett–Teller (BET) equation at a relative pressure range of 0.03–0.25. The content of acidic and basic functional groups was determined applying the Boehm titration method [18,19,20].

The specific surface area of examined materials was 950 m^2^/g for LB_AC and 1145 m^2^/g for MT_AC. Both activated carbons were mesoporous materials with an average pore diameter of approximately 5 nm. The total pore volume of LB_AC activated carbons was 1.10 cm^3^/g, and that of MT_AC activated carbons was 1.47 cm^3^/g. The total content of surface groups of material obtained from the lemon balm herb was 0.987 mmol/g, and the content of surface groups of material derived from the mint herb was 0.794 mmol/g. The tested activated carbons were characterized by a highly developed surface area and a high total pore volume in comparison to other materials described in the literature (Table 1).

Adsorption, stability, electrokinetic and aggregation measurements were performed at pH 3, at which the ionic polymers used demonstrated the highest affinity to both activated carbons’ surfaces—their adsorbed amounts were the largest. All of these measurements were performed at 25 °C in the following systems: LB_AC, LB_AC+PAA, LB_AC+PEI, LB_AC+PAA+PEI, MT_AC, MT_AC+PAA, MT_AC+PEI and MT_AC+PAA+PEI.

Anionic poly(acrylic acid) (PAA) (Fluka, Saint Louis, MO, USA) with an average molecular weight equal to 2000 Da is an anionic polymer containing carboxyl functional groups. At pH 3, it occurs in a coiled form due to minimal ionization; the dissociation degree of PAA functional groups is approximately 0.03 [26].

Polyethyleneimine (PEI) (Sigma Aldrich, Saint Louis, MO, USA) with an average molecular weight of 2000 Da is a cationic macromolecular substance (with amine functional groups). At pH 3, its polymer chain is completely dissociated and assumes the conformation characterized by significant expansion in the aqueous solution [27].

**Figure 1:**
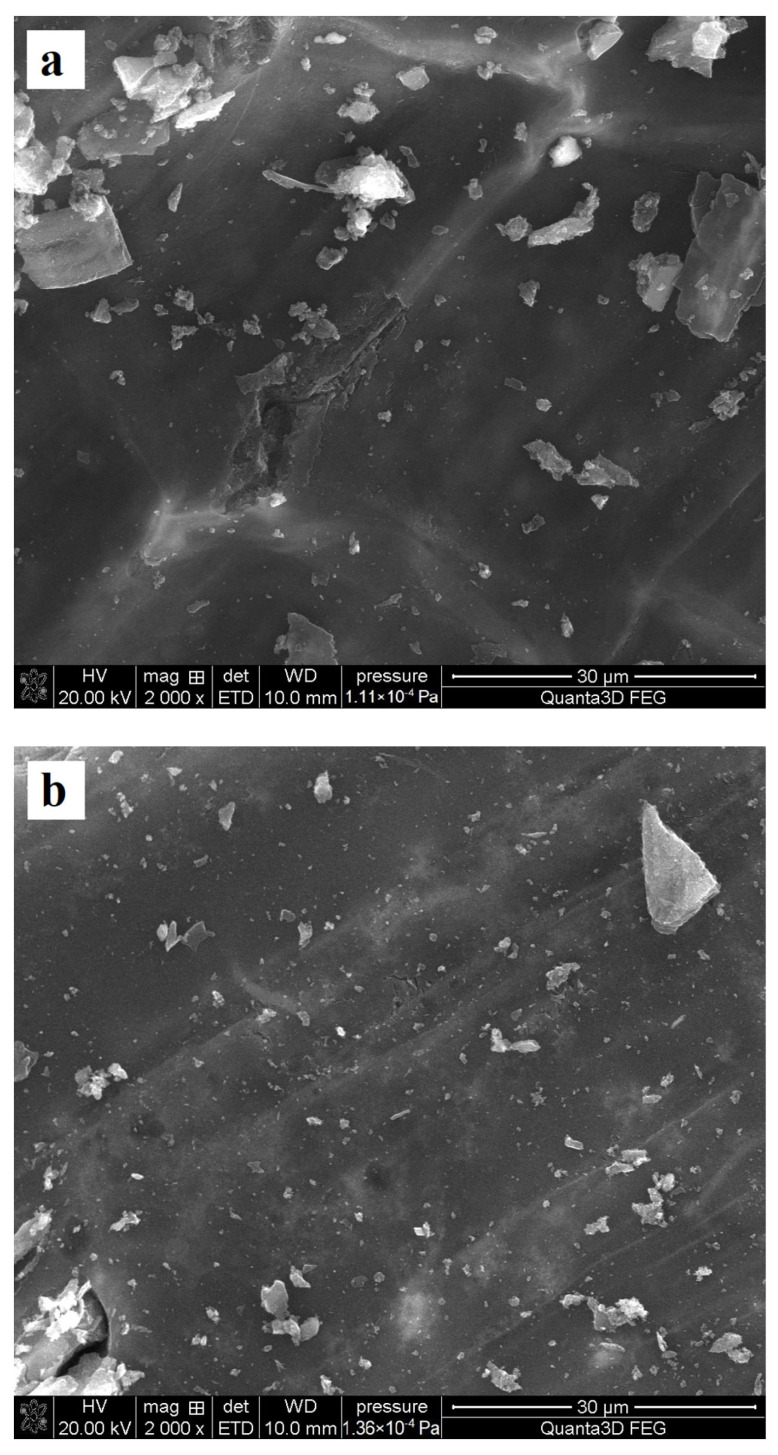
SEM images of MT_AC (**a**) and LB_AC (**b**) activated carbons.

**Figure 2:**
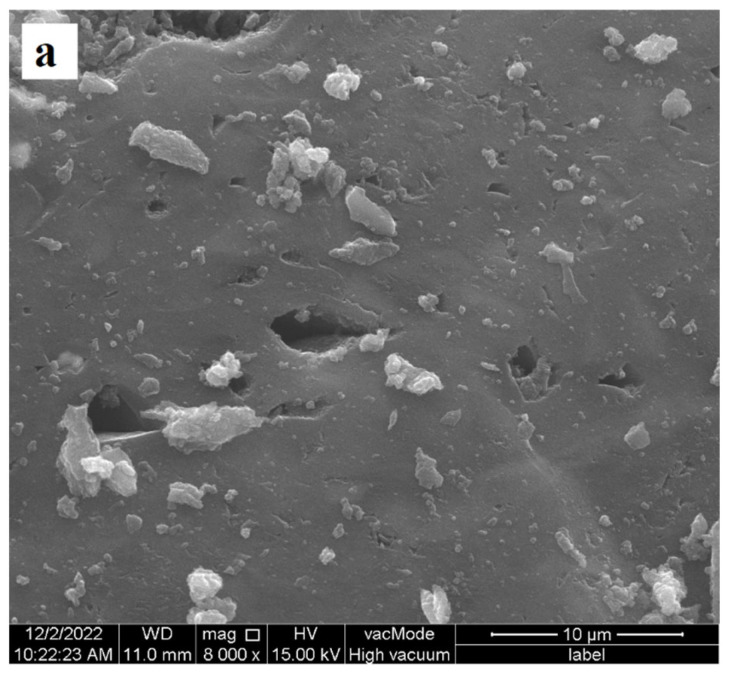

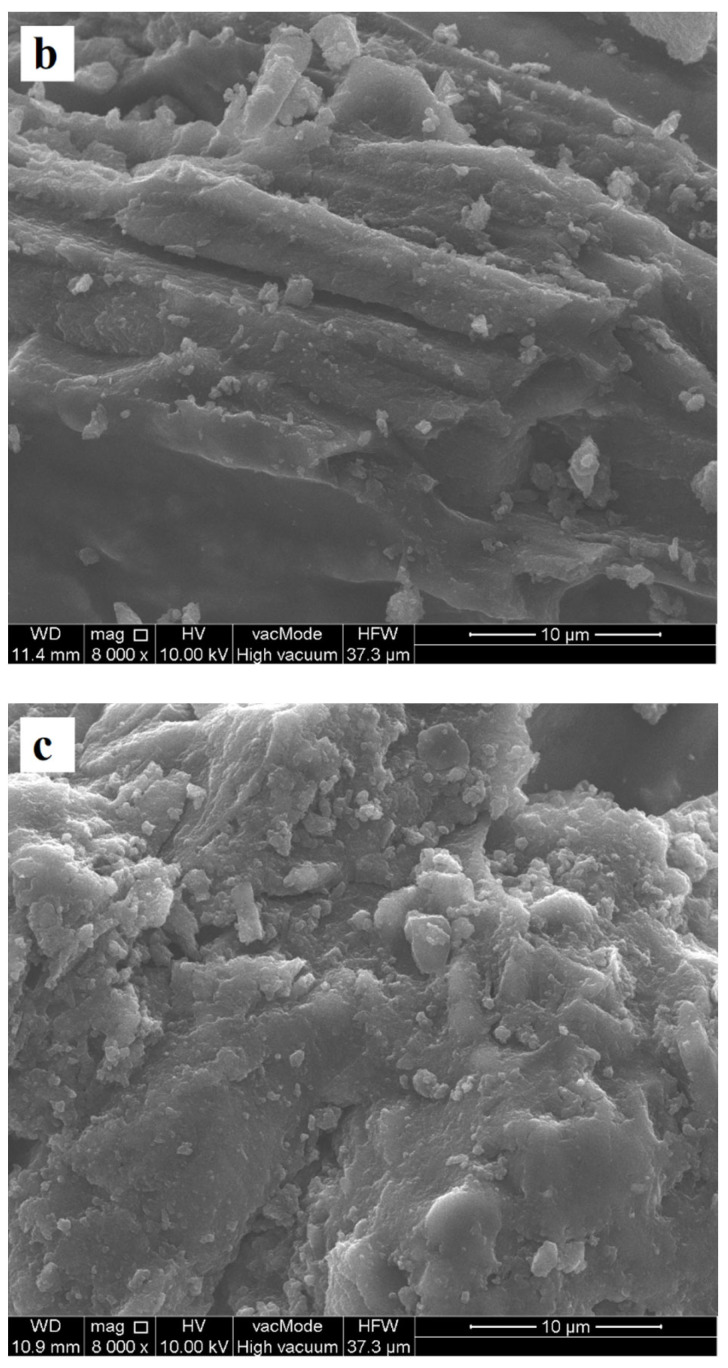
SEM images of MT_AC activated carbon without (**a**) as well as with PAA (**b**) and PEI (**c**) adsorbates.

The surface morphologies of activated carbons without and with adsorbed poly(acrylic acid) or polyethyleneimine were determined by applying the SEM technique (Quanta 250 FEG by FEI, Waltham, MA, USA; detector Octane Elect Plus by EDAX, Berwyn, IL, USA). SEM images of both activated carbons are presented in Figure 1, whereas Figure 2 shows the morphology of the MT_AC sample in the presence of polymers.

### 2.2. Surface Charge Density, Zeta Potential and Aggregate Sizes Determination

Determination of the surface charge density (σ_0_) of activated carbons particles without or with the selected adsorbates was performed by applying the potentiometric titration method. For this purpose, 50 cm^3^ of a suspension containing 0.025 g of activated carbon, 100 ppm of adsorbates and 0.001 mol/dm^3^ of NaCl supporting electrolyte was used. These measurements were performed in a thermostated Teflon vessel (RE 204 thermostat, Lauda Scientific, Lauda-Königshofen, Germany) in which glass and calomel electrodes (Beckman Instruments, Brea, CA, USA) were placed. Changes in the σ_0_ value as a function of solution pH were calculated with the computer program “Titr_v3” [28].

The zeta potential (ζ) and size of activated carbons particles aggregates (without and with the polymer/s) were determined using the following procedure. First, 200 cm^3^ of suspension containing the tested polymers with a concentration of 100 ppm, NaCl supporting electrolyte (0.001 mol/dm^3^) and 0.03 g of the solid was prepared. The system was subjected to the action of ultrasounds (XL 2020 ultrasonic head, Misonix, Farmingdale, NY, USA) for 3 min, and then the pH of the obtained sample was adjusted to 3. For this purpose, solutions of HCl and NaOH with concentrations of 0.1 mol/dm^3^ as well as a Φ360 pH meter (Beckman, Brea, CA, USA) were used. Zeta potential measurements were performed by applying the Doppler laser electrophoresis method and a Zetasizer Nano ZS (Malvern Instruments, Malvern, UK). This device facilitated measurement of the electrophoretic mobility of solid particles without, and covered with, polymeric adsorption layers. Based on obtained results, the zeta potential (ζ) was calculated using the Henry’s equation [29]. In turn, the average size of aggregates of tested solids was determined based on the static light scattering phenomenon. In the case of suspensions containing polymer/s, the macromolecular compound was added directly after the sonication process, and such samples were immediately subjected to appropriate measurement.

### 2.3. Adsorption Studies

Adsorption tests were performed at 25 °C for 24 h; 10 cm^3^ of suspension containing 0.1 g of activated carbon, 200 ppm of the appropriate polymer and 0.001 mol/dm^3^ of NaCl as the supporting electrolyte was used. Adsorption was carried out in single and binary systems of polymeric adsorbates. After the process was completed, solids were separated from solutions using the MPW 233e microcentrifuge (MPW Med. Instruments, Warsaw, Poland). The amounts of adsorbed polymers were determined using the static method based on the change in the adsorbate concentration in the solution before and after the process. The concentration of poly(acrylic acid) was determined based on its reaction with hyamine 1622, which resulted in the formation of a white-colored complex. The obtained compound absorbed light at a wavelength of 500 nm [30]. In the case of polyethyleneimine, a reaction with CuCl_2_ was used. This formed a blue-colored complex that absorbed light at a wavelength of 285 nm [31]. The UV–Vis spectrophotometer Carry 100 (Varian, Palo Alto, CA, USA) was used for absorbance measurements.

### 2.4. Stability Tests

**Figure 3:**
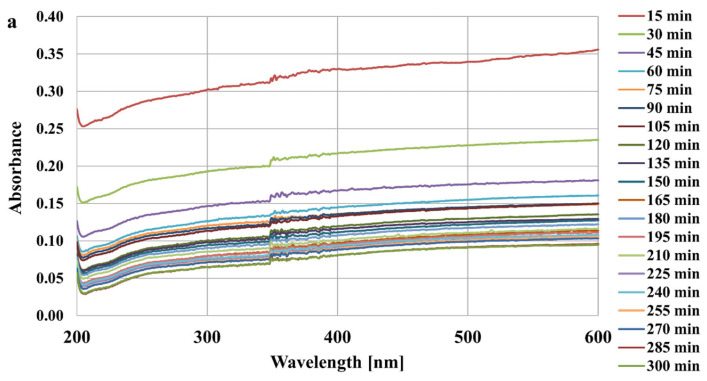

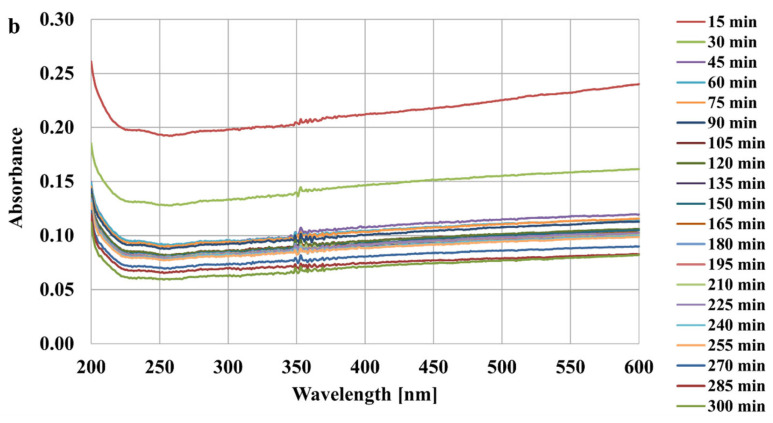
The absorbance of LB_AC (**a**) and MT_AC (**b**) activated carbons suspensions at different wavelengths.

The spectrophotometric method was applied for stability studies (spectrophotometer Carry 100, Varian, Palo Alto, CA, USA). For this purpose, 10 cm^3^ of suspension containing 0.01 g of activated carbon, 0.001 mol/dm^3^ of NaCl (supporting electrolyte) and 200 ppm of appropriate adsorbate was prepared. The pH of the sample was adjusted to 3 using HCl and NaOH solutions with concentrations of 0.1 mol/dm^3^ as well as a Φ360 pH meter (Beckman, Brea, CA, USA). The exemplary absorbance dependencies obtained for LB_AC and MT_AC activated carbons suspensions at different wavelengths are presented in Figure 3. The wavelength of 500 nm was chosen for further study, due to the lack of interferences and small changes in the recorded signal. The absorbance of tested suspensions was measured for 5 h at 15 min intervals.

## 3. Results and Discussion

### 3.1. Electrical Double-Layer Structure and Aggregation Properties of Activated Carbons in the Presence of Ionic Polymers

The surface charge density and zeta potential values of examined activated carbons at pH 3 are presented in Table 2. The surface charge density of the material obtained from the lemon balm was 4.52 μC/cm^2^, whereas the surface charge density of the material obtained from the mint herb was 2.54 μC/cm^2^. The presence of poly(acrylic acid) slightly decreased the surface charge density at pH 3. The attraction between positively charged adsorbents surfaces and the anionic polymer is favorable for PAA adsorption under such pH conditions. The presence of polyethyleneimine increased the surface charge density. The impact of PEI on the examined value was more noticeable due to the repulsion between positively charged polymeric macromolecules and the adsorbent surface. The polymeric chains were not fully adsorbed on the activated carbons surface in the form of train structures (by all their segments). Some amino groups present in the loop and tail structures of adsorbed chains were located in the by-surface area, which affected the surface charge density value. In the binary solution, an intermediate effect, in comparison to the single solution, was observed. The zeta potential of LB_AC activated carbon was 3.90 mV, whereas that of MT_AC activated carbon was −4.72 mV. These values are close to zero, which proved that the isoelectric points of both materials occured at a pH of approximately 3. Poly(acrylic acid) present in the solution caused decreases in zeta potential values, whereas in the system containing polyethyleneimine the zeta potential increased. This was caused by the charged polymeric functional groups of adsorbed macromolecules located on the border of the compact and diffusive parts of the electrical double layer (edl) formed at the activated carbon–solution interface [32]. In the binary solution, the greatest increase in the zeta potential was observed. At pH 3, PAA occurs in a coiled form, and can be adsorbed on the surface of activated carbon as well as inside its solid pores (due to PAA macromolecules small hydrodynamic radius of 0.77 nm), whereas the mean pore size of both adsorbents is approximately 5 nm [33]. PEI chains under these conditions assume considerably developed conformation and their adsorption is mostly limited to the outer surface of carbonaceous adsorbents. Due to the poly(acrylic acid) occupation of active sites on the solid surface, a greater amount of polyethyleneimine molecules remained in the slipping plane area, which resulted in an additional increase in the zeta potential value. The binary system was presumably the most stable among all tested suspensions because the higher the absolute value of the zeta potential, the greater the probability that the studied suspension will be stable [34].

**Figure 4:**
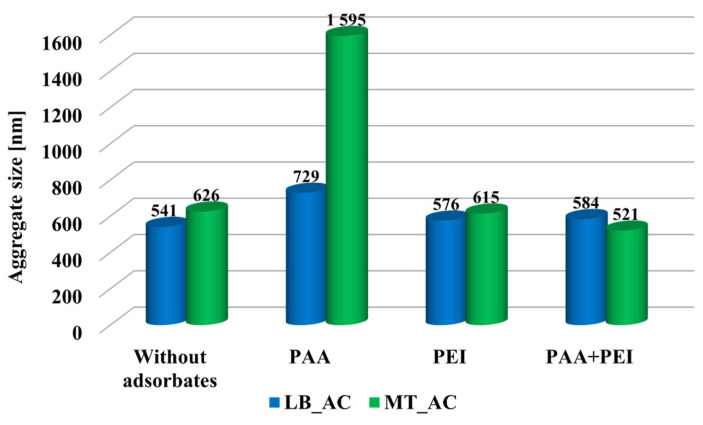
Sizes of LB_AC and MT_AC activated carbons aggregates at pH 3 (without adsorbates, as well as in single and binary adsorbate systems).

The presence of poly(acrylic acid) caused an increase in the size of the aggregates of both activated carbons (Figure 4). The adsorption of coiled PAA molecules induced changes in the surface charge density leading to the strongest attraction between adsorbent particles. The adsorption of negatively charged polymeric chains causes the neutralization of the positive surface charge of activated carbons. MT_AC activated carbon contains a smaller number of surface functional groups, which results in lower adsorption of poly(acrylic acid). In this case, the polymeric coils create a less compact adsorption layer and there is a possibility of bridging interactions occurrence. As a result, larger aggregates can be created, which, due to their loose structure, remain in the solution (the stabilization effect is observed). In suspensions containing PEI or both polymer adsorbates, no significant changes in the size of LB_AC and MT_AC aggregates were observed. Large aggregates usually flocculate faster; however, stable macroaggregates also exist [35]. Aggregates with a well-developed surface area can undergo sedimentation more slowly, due to the buoyancy forces of the solvent, and thus remain in the bulk solution for a long time.

### 3.2. Adsorption Layer Structure of Ionic Polymers on the Activated Carbons Surface

**Figure 5:**
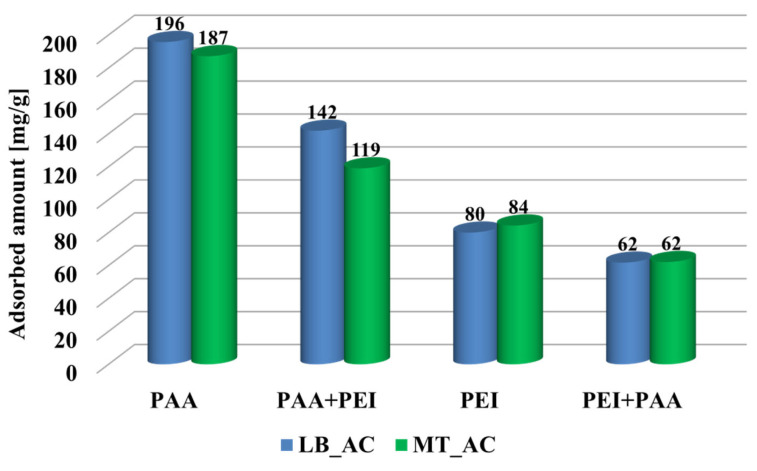
Adsorbed amounts of poly(acrylic acid) (PAA, PAA + PEI) and polyethyleneimine (PEI, PEI + PAA) on the surface of LB_AC and MT_AC activated carbons at pH 3 in single and binary adsorbates systems.

The anionic polymer showed greater affinity to the surface of LB_AC activated carbon (Figure 5). The higher content of functional surfaces groups for this material guaranteed the possibility of binding a larger number of macromolecule segments to the active sites of the solid. Polyethyleneimine was adsorbed in similar amounts on the surfaces of both adsorbents, as a result of its considerably developed conformation, which is less sensitive to the number of available active sites on the solid surface. The anionic polymer was adsorbed in a greater amount in comparison to the cationic one. It was related to a completely different polymers conformation at pH 3. PAA occurs in a coiled form and can be adsorbed both on the surface and in the pores of adsorbents, whereas developed PEI chains are mainly adsorbed only on the outer surface of activated carbons [17]. Moreover, the surface charge of the adsorbents at pH 3 was positive, which resulted in attraction between the anionic polymeric coils and the activated carbon particles. Such favorable electrostatic interactions resulted in the formation of a densely packed polymeric layer. In the case of PEI, the adsorption layer consisted of developed chains located perpendicularly to the solid surface. In the binary solutions, the adsorbed amounts of both polymers decreased due to competition between macromolecules with opposite ionic natures [36].

### 3.3. Stability Mechanisms of Activated Carbons Suspensions in the Presence of Ionic Polymers

**Figure 6:**
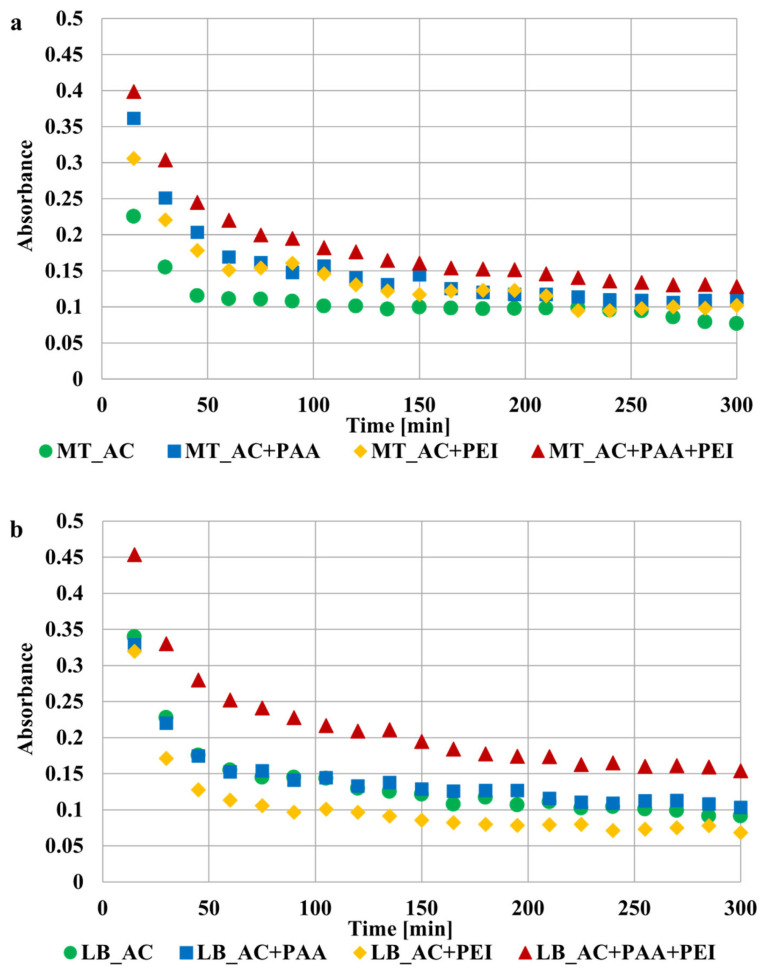
The stability of LB_AC (**a**) and MT_AC (**b**) activated carbons suspensions at pH 3 in single and binary adsorbate systems.

**Figure 7:**
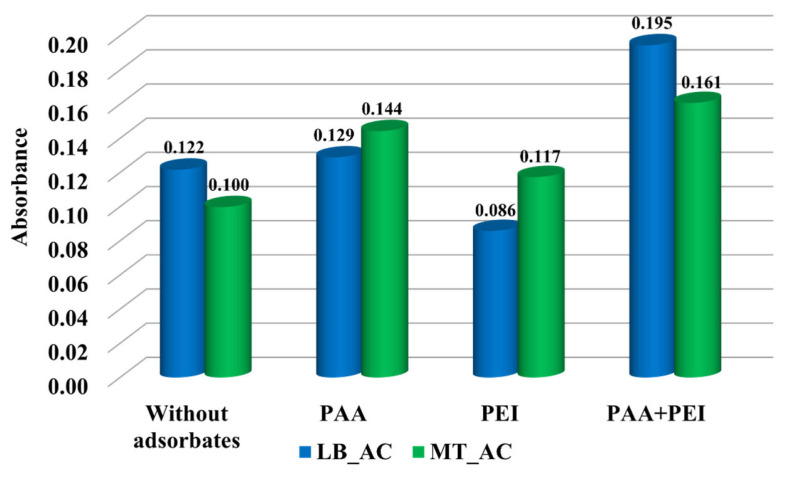
The absorbance of LB_AC and MT_AC activated carbons suspensions at pH 3 in single and binary adsorbate systems after 150 min.

**Figure 8:**
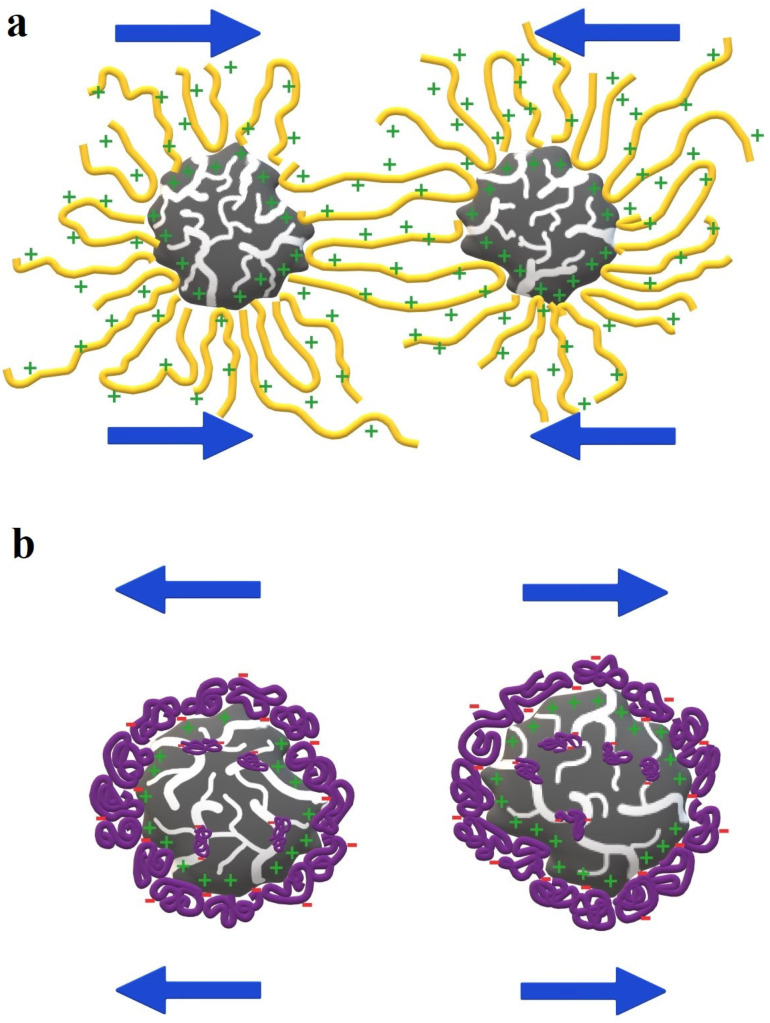

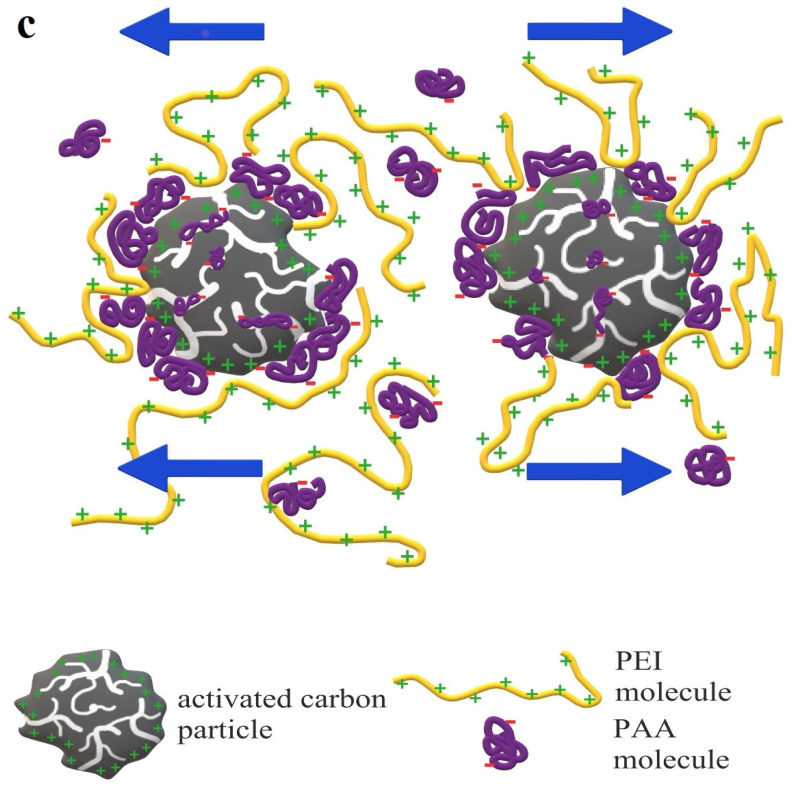
The major stabilization/destabilization mechanisms of PEI—the bridging flocculation (**a**), PAA—the electrosteric stabilization (**b**), and the binary system—the combined electrosteric and depletion stabilization (**c**).

Results of stability tests are presented in Figure 6 and Figure 7. The higher the absorbance value, the more stable the suspension (due to the presence of solid particles in the entire volume of the solution). The presence of poly(acrylic acid) had no significant effect on the stability of the LB_AC activated carbon suspension. The polymer was probably adsorbed most in the pores of the adsorbent, which did not significantly affect the interactions of solid particles. On the other hand, polyethyleneimine adsorption caused a noticeable deterioration in the stability of the system. The positively charged surface of the material obtained from the lemon balm caused the perpendicular adsorption of PEI cationic chains. Molecules were extended at considerable distances from the solid surface, which resulted in bridging flocculation by forming polymeric connections between neighboring particles (Figure 8a) [37]. In a different way, poly(acrylic acid) caused the stabilization of the MT_AC system due to electrosteric mechanisms occurrence (Figure 8b). Both activated carbons systems were the most stable in the simultaneous presence of PAA and PEI. All above-mentioned stabilization mechanisms occurred in binary systems. Additionally, depletion stabilization forces might appear (Figure 8c). Free chains that remained in the solution due to the lower adsorbed amount in the binary systems prevented colloidal particles from forming large aggregates and suspension destabilization [38].

## 4. Conclusions

The tested activated carbons were characterized by high adsorption capacities towards ionic polymers. The adsorbed amounts reached levels of approximately 190 mg/g for poly(acrylic acid) and approximately 80 mg/g for polyethyleneimine. The adsorption of polymers from their binary solutions decreased noticeably due to the competition of polymeric chains assuming completely different conformations in tested systems at pH 3. Ionic polymers had a strong impact on the activated carbons suspensions’ stability. Their presence also affected the surface charge density, zeta potential and aggregates sizes formed by solid particles. The most perpendicular adsorption of significantly developed PEI chains on the LB_AC activated carbon surface caused the destabilization of suspension via bridging flocculation. On the other hand, steric and electrosteric stabilization occurred in systems containing MT_AC material and ionic polymers. Binary systems were the most stable due to the occurrence of an additional stabilization mechanism coming from depletion forces. The competition between polyethyleneimine and poly(acrylic acid) macromolecules resulted in some polymeric chains remaining in the solution, which noticeably reduced the flocculation tendency.

## Figures and Tables

**Table 1:** Comparison of the preparation procedure and textural parameters of activated carbons obtained as a result of chemical activation of biomass.

Precursor	Preparation Conditions	Surface Area [m^2^/g]	Total Pore Volume [cm^3^/g]	Reference
Lemon balm herb	Impregnation with H_3_PO_4_, two stage heating program (200 °C, 500 °C)	950	1.10	Present study
Mint herb	Impregnation with H_3_PO_4_, two stage heating program (200 °C, 500 °C)	1145	1.47	Present study
Peanut hulls	Chemical activation with ZnCl_2_, heating for 6 h at temp. range 300–750 °C	420	0.17	[21]
Chestnut wood	Impregnation with H_3_PO_4_, heating at 500 °C for 4 h	783	0.44	[22]
Pineapple peels	Carbonized at 700 °C, activation with KOH in microwave oven	1006	0.59	[23]
Pineapple peels	Carbonized at 700 °C, activation with K_2_CO_3_ in microwave oven	680	0.45	[23]
Tomato processing solid waste	Impregnation with ZnCl_2_, carbonization at 600 °C, for 1 h	1093	1.57	[24]
Cotton cake	Impregnation with H_3_PO_4_, heating at 450 °C for 2 h	584	0.30	[25]
Shea cake	Impregnation with H_3_PO_4_, heating at 450 °C for 2 h	1148	0.61	[25]

**Table 2:** The surface charge density and zeta potential of LB_AC and MT_AC activated carbons at pH 3 without adsorbates as well as in single and binary adsorbates systems.

System	Surface Charge Density [μC/cm^2^]		Zeta Potential [mV]	
	LB_AC	MT_AC	LB_AC	MT_AC
Without adsorbates	4.52	2.54	3.90	−4.72
PAA	2.52	1.47	−3.00	−4.19
PEI	14.33	14.59	26.02	19.77
PAA + PEI	11.23	3.40	32.08	33.42

## Data Availability

Data are contained within the article.
